# Supplementary material for: Improved molecular characterization of the Klebsiella oxytoca complex reveals the prevalence of the kleboxymycin biosynthetic gene cluster
Source: Microb Genom. 2021 Jun 18;7(6):000592. doi: 10.1099/mgen.0.000592 (PMC8461473; doi:10.1099/mgen.0.000592)
Supplement: Supplementary material 1 [file mgen-7-0592-s001.pdf]

SUPPLEMENTARY MATERIAL ASSOCIATED WITH SHIBU *ET AL.*

Shibu P, McCuaig F, McCartney AL, Kujawska M, Hall LJ, Hoyles L. Improved molecular characterization of the *Klebsiella oxytoca* complex reveals the prevalence of the kleboxymycin biosynthetic gene cluster.

METHODS

Phenotypic characterization of clinical isolates. Once in the laboratory, API 20E strips (bioMerieux) were used to confirm the identities of the clinical isolates as *K. oxytoca*, following manufacturer's instructions.

Antimicrobial susceptibility testing. The three isolates were screened on Mueller–Hinton agar for possible carbapenemase production following UK national guidelines (1). This involved testing all presumptive isolates against 18 antimicrobials (amikacin, amoxycillin, augmentin, aztreonam, cefotaxime, cefoxitin, ceftazidime, cefuroxime, ciprofloxacin, colistin, ertapenem, gentamicin, meropenem, tazocin, temocillin, tigecycline, tobramycin, trimethoprim) following the EUCAST disc diffusion method. Control organisms used to monitor test performance of the antimicrobials were *Escherichia coli* ATCC 25922 and *Staphylococcus aureus* ATCC 29213. Zone sizes were read using calibrated callipers and interpreted as sensitive or resistant by referring to the EUCAST breakpoint guidelines (2).

Carbapenemase confirmatory tests were performed on all three isolates as they were found to be resistant to the indicator carbapenem ertapenem. The isolates were further tested with ertapenem E-strips (Launch) to ascertain the Minimum Inhibitory Concentration (MIC). After incubation for 24 h at 30–35 °C the MIC gradients were read against the EUCAST breakpoint guidelines and those showing MICs >0.12 µg/mL were considered resistant to ertapenem (2).

Extraction of DNA. Isolates were plated onto MacConkey agar no. 3 (Oxoid) and incubated aerobically and overnight at 37 °C. On three separate passages, a single colony of each isolate was picked and streaked out. After the third passage, DNA was extracted from a loopful of cells using the Gentra PureGene Qiagen DNA extraction kit (Qiagen). DNA quality was assessed by agarose gel electrophoresis, and quantified using the Nanodrop instrument.

Whole-genome sequencing, assembly and annotation. Extracted DNA was frozen at -20 °C and sent to the Quadram Institute Bioscience, Norwich for library preparation and sequencing. Samples

were run on an Illumina Nextseq500 instrument using a Mid Output Flowcell [NSQ® 500 Mid Output KT v2 (300 CYS)] following Illumina's recommended denaturation and loading procedures, which included a 1% PhiX spike-in (PhiX Control v3). Data were uploaded to Basespace, where the raw data were converted to eight fastq files for each sample (four for R1, four for R2), which were subsequently concatenated to produce one R1 and one R2 file per strain. Sequence data were quality checked using fastqc v0.11.8 (https://www.bioinformatics.babraham.ac.uk/projects/fastqc/): no adaptor or over-represented sequences were present. Data were trimmed using trimmomatic 0.39 (SLIDINGWINDOW:5:20 MINLEN:50) (3), and paired reads retained. Genomes were assembled using the trimmed paired reads with SPAdes v3.13.0 (default settings) (4). Completeness and contamination of the three genomes was assessed using CheckM v1.0.18 (5). Gene predictions and annotations were completed using Prokka v1.14.5 (default settings) (6). The data have been deposited with links to BioProject accession number PRJNA562720 in the NCBI BioProject database, and under accession numbers VTQC00000000, VTQB00000000 and VTQA00000000.

Identification of antimicrobial and virulence genes. The three draft genomes were uploaded to the *Klebsiella oxytoca* MLST website (https://pubmlst.org/koxytoca/) hosted by the University of Oxford (7) to determine allele number against previously defined house-keeping genes (*rpoB*, *gapA*, *mdh*, *pgi*, *phoE*, *infB* and *tonB*). Kleborate (8,9) and Kaptive (http://kaptive.holtlab.net) were used to attempt to identify capsular (K) and O antigen types (8–10). Presence of antibiotic-resistance genes within strains was determined by BLASTP analysis of amino acid sequences of predicted genes within genomes against the Comprehensive Antibiotic Resistance Database (CARD) database v3.0.7 (downloaded 27 July 2019; protein homolog dataset) (11); only strict and perfect matches with respect to CARD database coverage and bit-score cut-off recommendations are reported, to reduce the potential for reporting false-positive results. Virulence genes were identified by BLASTP of genome amino acid sequences against the Virulence Factors of Pathogenic Bacteria Database (VFDB; 'core dataset' downloaded 27 July 2019) (12); results are reported for ≥70 % identity and 90 % query coverage (13).

***bla*$_{\mathrm{OXY}}$ analysis.** *bla*$_{\mathrm{OXY}}$ protein sequences available from the Institut Pasteur MLST and Whole Genome MLST Databases were downloaded on 6 February 2021. They were used to create a BLASTP database against which Prodigal-annotated (Prodigal v2.6.2; (14)) *K. oxytoca* complex genes were searched. *bla*$_{\mathrm{OXY}}$ protein sequences were used to create a MSA (CLUSTAL W, BLOSUM matrix), from which a neighbour-joining tree was generated.

Phylogenetic placement of the isolates within the *K. oxytoca* complex. PhyloPhlAn v0.99 (15) was used to determine phylogenetic placements of the isolates within the *K. oxytoca* complex. PhyloPhlAn identifies hundreds of conserved (core) proteins from a given genomic dataset and uses them to build a complete high-resolution phylogeny.

RESULTS

Antimicrobial susceptibility of the isolates

PS_Koxy1, PS_Koxy2 and PS_Koxy4 were shown to be isolates of *K. oxytoca* by phenotypic testing (API 20E profile 5245773, 97.8 % identity). The strains were all found to be resistant to amoxicillin (zone diameter <14 mm), augmentin (<19 mm), aztreonam (<21 mm), cefotaxime (<17 mm), cefoxitin (<19 mm), ceftazidime (<19 mm), cefuroxime (<18 mm), ciprofloxacin (<19 mm), ertapenem (<22 mm), gentamicin (<14 mm), tazocin (<17 mm), temocillin (<19 mm), tobramycin (<14 mm) and trimethoprim (<14 mm), and sensitive to amikacin (>18 mm), colistin (MIC <2 µg/mL), meropenem (>22 mm) and tigecycline (>18 mm). Ertapenem resistance was confirmed by E-test (had an MIC > 0.12 µg/mL), as ertapenem and meropenem are used as an indicator antibiotic for the detection of carbapenemase.

Genotypic characterization of the three clinical isolates

We generated draft genome sequence data for PS_Koxy1, PS_Koxy2 and PS_Koxy4 to accurately identify the strains and provide us with genomic data that would be useful in our future phage studies (**Supplementary Table A**).

Supplementary Table A. Sequencing summary statistics for the three clinical isolates characterized in this study (all 60× coverage)

Isolate	Size (bp)	No. contigs	N50	CDS	Completeness, contamination*
PS_Koxy1	6,271,778	135	170,962	5,948	100 %, 0.30 %
PS_Koxy2	6,275,379	111	172,981	5,946	100 %, 0.30 %
PS_Koxy4	6,294,880	112	146,343	5,978	100 %, 0.30 %

*Determined using CheckM v1.0.18.

Antimicrobial resistance (AMR) gene profiles of the three clinical isolates

All three isolates encoded $bla_{\text{OXY1-8}}$, showing they belonged to *K. michiganensis*, not *K. oxytoca* (**Supplementary Figure 1**). Phylogenetic analyses of the isolates with representatives of the *K. oxytoca* complex confirmed their affiliation with *K. michiganensis* (**Supplementary Figure 2**). The protein sequences encoded by the strains' genomes were compared against the CARD database (11). Presence of the β-lactamase with carbapenemase activity GES-5 (100 % identity, bit-score 591 – perfect CARD match) was confirmed, so too was that of the extended spectrum β-lactamase (ESBL) CTX-M-15 (100 % identity, bit-score 593 – perfect CARD match) (**Supplementary Figure 3**) (16). PS_Koxy1, PS_Koxy2 and PS_Koxy4 also encoded SHV-66 (99.65 % identity, bit-score 580 – strict CARD match). In addition to the β-lactamases GES-5, CTX-M-15 and SHV-66, PS_Koxy1, PS_Koxy2 and PS_Koxy4 encoded several other antibiotic-resistance genes (**Supplementary Figure 3a**), some reported as rare (e.g. *acrB*, *acrD*, *emrB*, *mdtB*, *mdtC*) in *K. oxytoca* genomes by CARD while others were common (e.g. *baeR*, *fosA5*, CRP, *marA*) (**Supplementary Table B**). Furthermore, PS_Koxy1 encoded AAC(6')-Ib7; PS_Koxy1 and PS_Koxy2 encoded *tet*(*A*); PS_Koxy2 and PS_Koxy4 encoded QnrB1 (**Supplementary Figure 3a**).

PS_Koxy1, PS_Koxy2 and PS_Koxy4 were resistant to the β-lactam antibiotics amoxicillin and aztreonam, augmentin and tazocin (both containing a β-lactam antibiotic and β-lactamase inhibitor), the cephalosporins cefotaxime, cefoxitin, ceftazidime and cefuroxime, the fluoroquinolone ciprofloxacin, the carbapenem ertapenem, the aminoglycosides gentamicin and tobramycin, the

carboxypenicillin temocillin, and the antifolate antibacterial trimethoprim. They were sensitive to amikacin (aminoglycoside), colistin (polymyxin), meropenem (carbapenem) and tigecycline (glycylcycline). *bla*$_{OXA-1}$ is frequently associated with *bla*$_{CTX-M-15}$, making isolates resistant to β-lactam–β-lactamase inhibitor combinations (19): all three strains were resistant to augmentin (amoxicillin/clavulanic potassium) and tazocin (piperacillin/tazobactam), with neither OXA-1 nor CTX-M-15 considered common in *K. oxytoca* genomes (**Supplementary Figure 3b**, **Supplementary Table B**). The strains carried a range of plasmid-encoded enzymes [AAC(6')-Ib7, *aadA*, APH(3'')-Ib, APH(6)-Id, AAC(3)-IIe] conferring resistance to gentamicin and tobramycin, though they remained sensitive to amikacin.

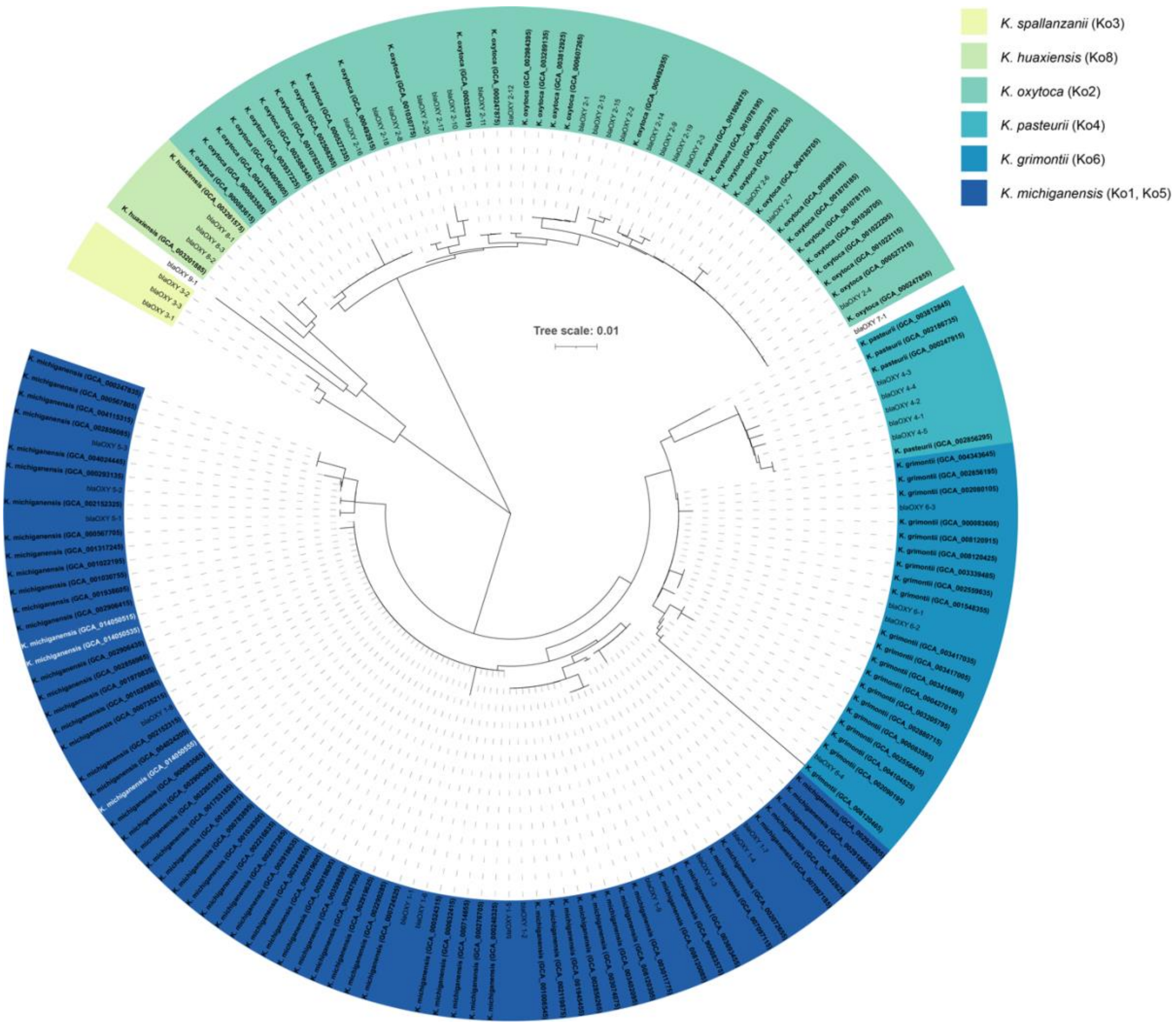

Supplementary Figure 1. *bla*$_{\text{OXY}}$ gene analysis shows PS_Koxy1, PS_Koxy2 and PS_Koxy4 (in white text) belong to phylogroup Ko1 and, therefore, are affiliated with *K. michiganensis*. The phylogenetic tree (neighbour joining, Jukes Cantor) was generated using *bla*$_{\text{OXY}}$-encoding protein sequences from the genomes of the three clinical isolates and publicly available *K. oxytoca* complex genomes (**Supplementary Table 1**). Scale bar, average number of amino acid substitutions per position.

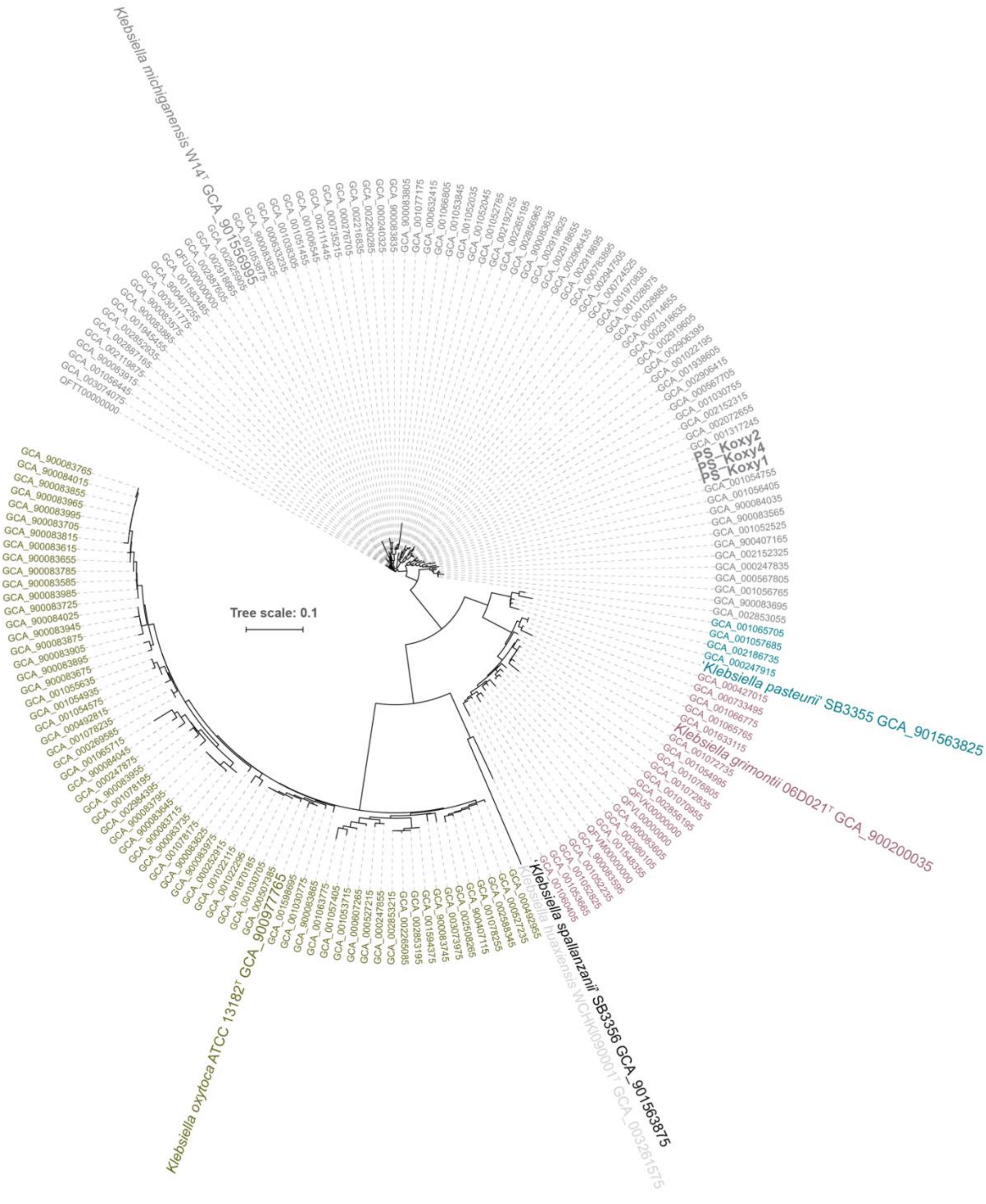

Supplementary Figure 2. Phylogenetic tree showing the relationship of the three GES-5-positive clinical isolates (PS_Koxy1, PS_Koxy2, PS_Koxy4) and the 167 strains included in our previous study (13), plus reference strains of species of the *K. oxytoca* complex (17). The tree was generated using PhyloPhlAn v0.99 and 380 protein-encoding sequences conserved across all genomes. Scale bar, average number of amino acid substitutions per position.

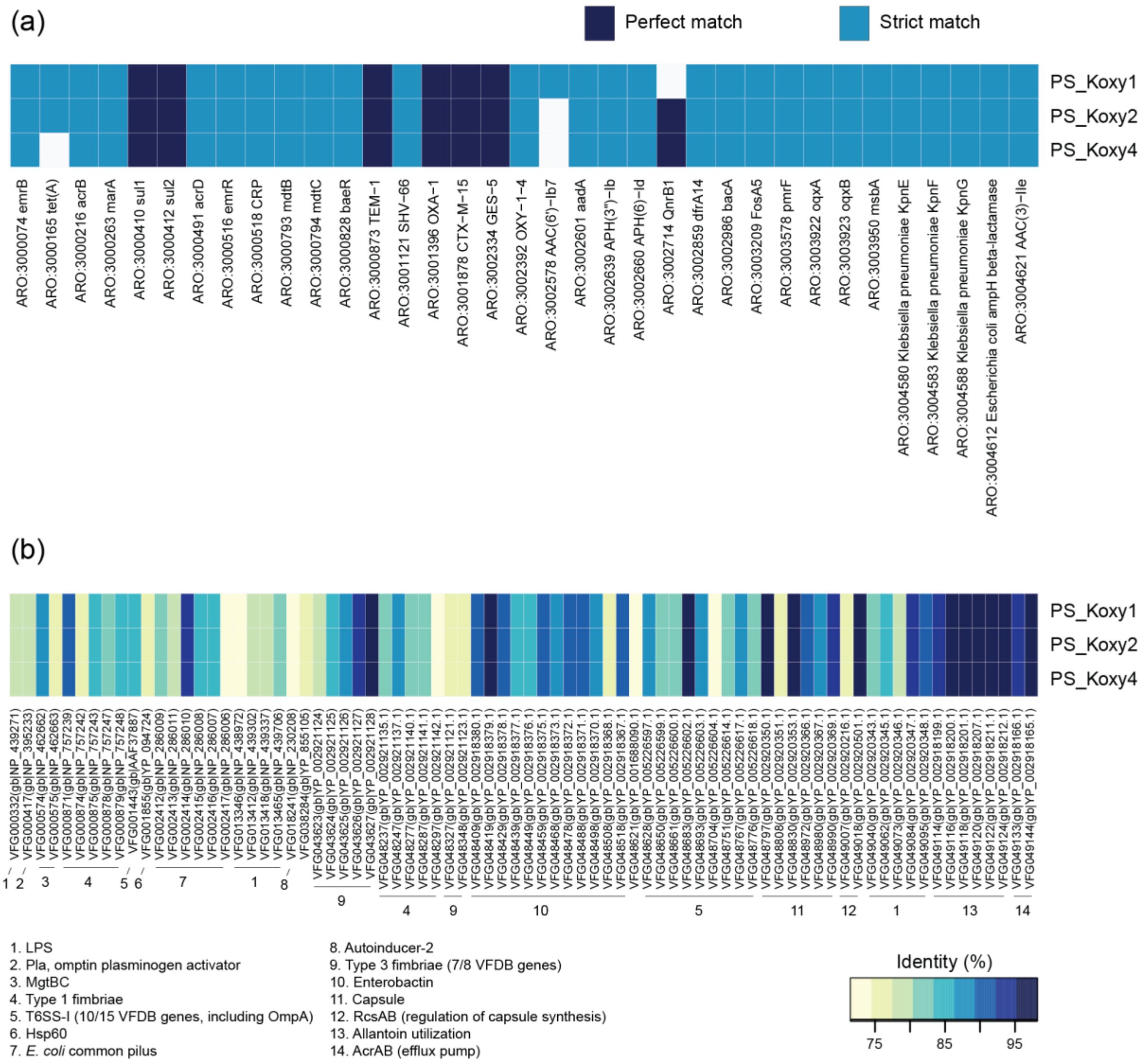

Supplementary Figure 3. Antimicrobial resistance (a) and virulence factor (b) genes detected in the genomes of the GES-5-positive *K. michiganensis* strains. (a) Protein sequences encoded within the strains' genomes were compared against sequences in CARD (11). Strict CARD match, not identical but the bit-score of the matched sequence is greater than the curated BLASTP bit-score cut-off; perfect CARD match, 100 % identical to the reference sequence along its entire length. (b) Protein sequences encoded within the strains' genomes were compared against sequences in VFDB (12). Only BLASTP results for proteins sharing >70 % identity and 90 % query coverage with VFDB protein sequences are shown.

Supplementary Table B. Summary of information for CARD genes found in *K. michiganensis* PS_Koxy1, PS_Koxy2 and PS_Koxy4

CARD data from analyses of 257 *K. oxytoca* complex genomes (https://card.mcmaster.ca/prevalence).

CARD Prevalence 3.0.7 is based on sequence data acquired from NCBI on 7 May 2020.

**ARO: accession**	**Name**	**Definition**	**Prevalence (%) in *K. oxytoca****
3000074	*emrB*	Translocase in the *emrB*-TolC efflux protein in *E. coli*. It recognises substrates including carbonyl cyanide *m*-chlorophenylhydrazone, nalidixic acid and thioloactomycin.	0.78
3000165	*tet(A)*	Tetracycline efflux pump found in many species of Gram-negative bacteria.	5.84
3000216	*acrB*	Protein subunit of AcrA-AcrB-TolC multidrug efflux complex. AcrB functions as a heterotrimer which forms the inner membrane component and is primarily responsible for substrate recognition and energy transduction by acting as a drug/proton antiporter.	0.78
3000263	*marA*	In the presence of antibiotic stress, *E. coli* overexpresses the global activator protein MarA, which besides inducing MDR efflux pump AcrAB, also down-regulates synthesis of the porin OmpF.	98.05
3000410	*sul1*	Sulfonamide resistant dihydropteroate synthase of Gram-negative bacteria. It is linked to other resistance genes of class 1 integrons.	13.23
3000412	*sul2*	Sulfonamide resistant dihydropteroate synthase of Gram-negative bacteria, usually found on small plasmids.	2.72
3000491	*acrD*	Aminoglycoside efflux pump expressed in *E. coli*. Its expression can be induced by indole, and is regulated by *baeRS* and *cpxAR*.	0.78
3000516	*emrR*	EmrR is a negative regulator for the EmrAB-TolC multidrug efflux pump in *E. coli*. Mutations lead to EmrAB-TolC overexpression.	99.61
3000518	CRP	CRP is a global regulator that represses MdtEF multidrug efflux pump expression.	100
3000793	*mdtB*	MdtB is a transporter that forms a heteromultimer complex with MdtC to form a multidrug transporter. MdtBC is part of the MdtABC-TolC efflux complex.	0.39
3000794	*mdtC*	MdtC is a transporter that forms a heteromultimer complex with MdtB to form a multidrug transporter. MdtBC is part of the MdtABC-TolC efflux complex. In the absence of MdtB, MdtC can form a homomultimer complex that results in a functioning efflux complex with a narrower drug specificity.	0.39
3000828	*baeR*	BaeR is a response regulator that promotes the expression of MdtABC and AcrD efflux complexes.	99.22
3000873	TEM-1	TEM-1 is a broad-spectrum beta-lactamase found in many Gram-negative bacteria. Confers resistance to penicillins and first generation cephalosphorins.	5.45
3001121	SHV-66	SHV-66 is an extended-spectrum beta-lactamase found in *K. pneumoniae*.	ND*
3001396	OXA-1	OXA-1 is a beta-lactamase found in *E. coli*.	1.95
3001878	CTX-M-15	CTX-M-15 is a beta-lactamase found in the family *Enterobacteriaceae*.	0.78
3002334	GES-5	GES-5 is a beta-lactamase found in the family *Enterobacteriaceae*.	ND
3002392	OXY-1-4	OXY-1-4 is a beta-lactamase found in *K. oxytoca*.	3.11
3002578	AAC(6’)-Ib7	AAC(6')-Ib7 is a plasmid-encoded aminoglycoside acetyltransferase in *Enterobacter cloacae* and *Citrobacter freundii*.	ND
3002601	*aadA*	ANT(3")-Ia is an aminoglycoside nucleotidyltransferase gene encoded by plasmids, transposons, integrons in	8.95

ARO: accession	Name	Definition	Prevalence (%) in *K. oxytoca**
		Enterobacteriaceae, *Acinetobacter baumannii*, *Pseudomonas aeruginosa* and *Vibrio cholerae*.	
3002639	APH(3’’)-Ib	APH(3")-Ib is an aminoglycoside phosphotransferase encoded by plasmids, transposons, integrative conjugative elements and chromosomes in *Enterobacteriaceae* and *Pseudomonas* spp.	4.67
3002660	APH(6)-Id	APH(6)-Id is an aminoglycoside phosphotransferase encoded by plasmids, integrative conjugative elements and chromosomal genomic islands in *K. pneumoniae*, *Salmonella* spp., *E. coli*, *Shigella flexneri*, *Providencia alcalifaciens*, *Pseudomonas* spp., *V. cholerae*, *Edwardsiella tarda*, *Pasteurella multocida* and *Aeromonas bestiarum.*	5.45
3002714	QnrB1	Plasmid-mediated quinolone resistance protein found in *K. pneumoniae*.	0.39
3002859	*dfrA14*	Integron-encoded dihydrofolate reductase found in *E. coli.*	5.45
3002986	*bacA*	BacA recycles undecaprenyl pyrophosphate during cell wall biosynthesis, which confers resistance to bacitracin.	0.39
3003209	*fosA5*	Fosfomycin resistance gene isolated from clinical strain of *E. coli* E265. It is susceptible to amikacin, tetracycline and imipenem, and resistant to sulphonamide, cephalosporins, gentamicin, ciprofloxacin, chloramphenicol and streptomycin.	93.39
3003578	*pmrF*	Required for the synthesis and transfer of 4-amino-4-deoxy-L-arabinose to Lipid A, which allows Gram-negative bacteria to resist the antimicrobial activity of cationic antimicrobial peptides and antibiotics such as polymyxin.	0.39
3003922	*oqxA*	RND efflux pump conferring resistance to fluoroquinolone.	91.83
3003923	*oqxB*	RND efflux pump conferring resistance to fluoroquinolone.	1.56
3003950	*msbA*	Multidrug resistance transporter homolog from *E. coli* and belongs to a superfamily of transporters that contain an adenosine triphosphate (ATP) binding cassette (ABC) which is also called a nucleotide-binding domain (NBD). MsbA is a member of the MDR-ABC transporter group by sequence homology. MsbA transports lipid A, a major component of the bacterial outer cell membrane, and is the only bacterial ABC transporter that is essential for cell viability.	98.83
3004580	*K. pneumoniae* KpnE	KpnE subunit of KpnEF resembles EbrAB from *E. coli*. Mutation in KpnEF resulted in increased susceptibility to cefepime, ceftriaxon, colistin, erythromycin, rifampin, tetracycline, and streptomycin as well as enhanced sensitivity toward sodium dodecyl sulfate, deoxycholate, dyes, benzalkonium chloride, chlorhexidine and triclosan.	98.83
3004583	*K. pneumoniae* KpnF	KpnF subunit of KpnEF resembles EbrAB from *E. coli*. Mutation in KpnEF resulted in increased susceptibility to cefepime, ceftriaxon, colistin, erythromycin, rifampin, tetracycline, and streptomycin as well as enhanced sensitivity toward sodium dodecyl sulfate, deoxycholate, dyes, benzalkonium chloride, chlorhexidine and triclosan.	99.22
3004588	*K. pneumoniae* KpnG	KpnG consists of ~390 residues and resembles EmrA of *E. coli*. Disruption of the pump components KpnG-KpnH significantly decrease resistance to azithromycin, ceftazidime, ciprofloxacin, ertapenem, erythromycin, gentamicin, imipenem, ticarcillin, norfloxacin, polymyxin-B, piperacillin, spectinomycin, tobramycin and streptomycin.	99.22
3004612	*E. coli ampH*	AmpH is a class C ampC-like beta-lactamase and penicillin-binding protein identified in *E. coli*.	99.22
3004621	AAC(3)-IIe	Plasmid-encoded aminoglycoside acetyltransferase in *E. coli*.	1.95

*ND, no data.

(a)

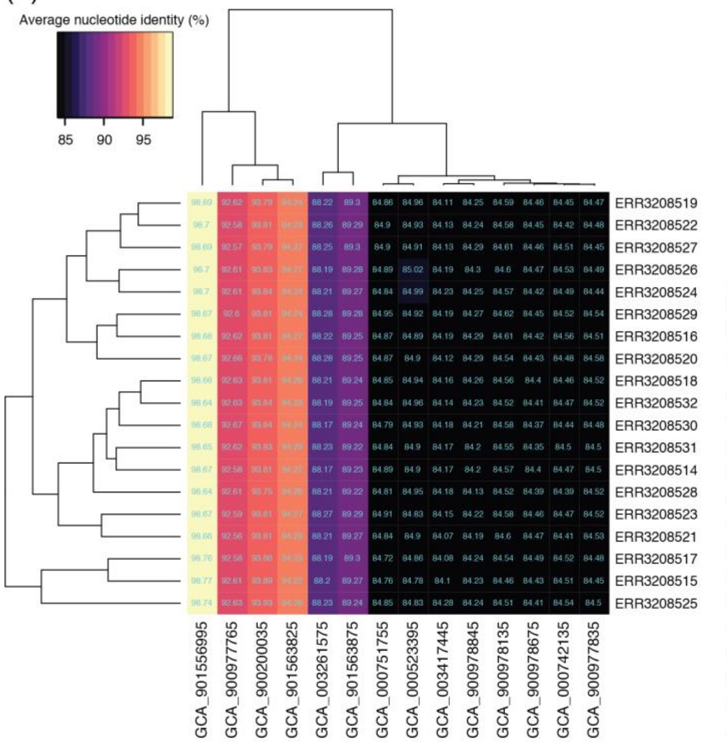

(b)

SRA accession	Size (bp)	No. contigs	N50	CDS
ERR3208514	6,302,966	335	114,717	5,880
ERR3208515	6,262,953	328	118,337	5,837
ERR3208516	6,287,539	341	124,824	5,862
ERR3208517	6,450,015	326	124,100	6,110
ERR3208518	6,317,731	331	118,589	5,901
ERR3208519	6,323,285	334	124,824	5,900
ERR3208520	6,323,567	341	114,717	5,898
ERR3208521	6,325,218	336	122,308	5,900
ERR3208522	6,329,222	336	124,824	5,911
ERR3208523	6,326,723	364	124,861	5,898
ERR3208524	6,315,651	362	103,560	5,896
ERR3208525	6,241,566	312	106,994	5,818
ERR3208526	6,329,241	362	114,717	5,904
ERR3208527	6,330,129	361	114,398	5,904
ERR3208528	6,322,073	380	114,717	5,899
ERR3208529	6,346,312	543	105,659	5,896
ERR3208530	6,309,195	402	114,398	5,887
ERR3208531	6,330,172	381	120,681	5,907
ERR3208532	6,319,562	337	124,861	5,900

(c)

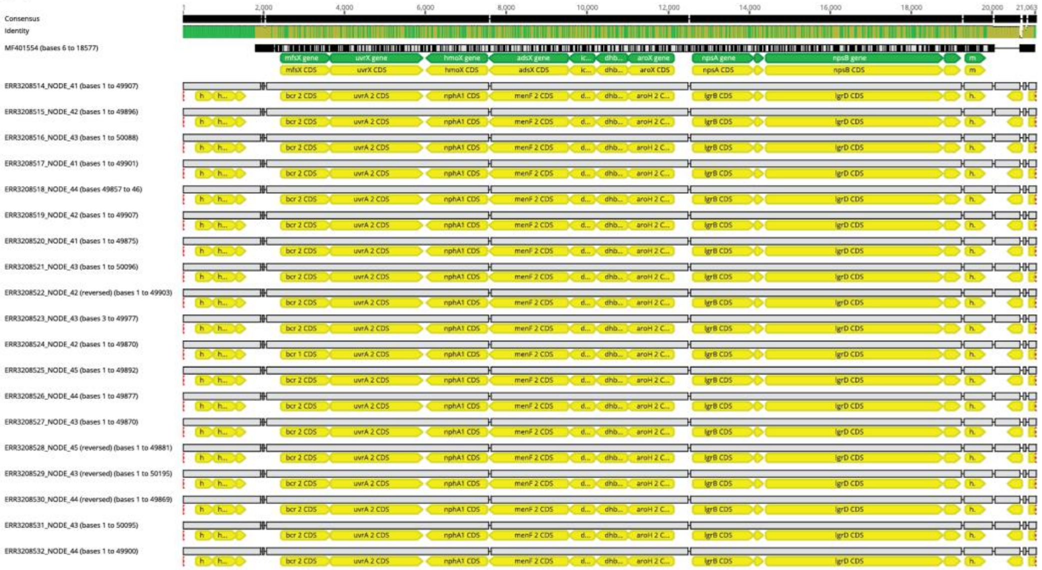

Supplementary Figure 4. Species identification of ST138 isolates described by Ellington *et al.* (18) and detection of the kleboxymycin BGC within their genomes. (a) Bidirectional clustered heatmap showing that all ST138 isolates described recently are *K. michiganensis*, not *K. oxytoca*, sharing 98.64–98.77 % ANI with GCA_901556995 (*K. michiganensis* W14^{T}). (b) Assembly statistics for the genomes assembled in this study with the Sequence Read Archive (SRA) accession numbers associated with the raw data. (c) The kleboxymycin BGC is present in all 19 of the *K. michiganensis* ST138 isolates of Ellington *et al.* (18). The image (alignment view) was generated via the progressiveMauve algorithm plugin of Geneious Prime v2019.2.1 (default settings, full alignment). Prokka-assigned gene annotations have been left for the *de novo*-assembled genomes. Consensus identity is the mean pairwise nucleotide identity over all pairs in the column: green, 100 % identity; greeny-brown, at least 30 % and under 100 % identity; red, below 30 % identity.

ERR3208533 was also assembled but was found to be *K. oxytoca* and lacking the kleboxymycin BGC (data not shown, but assembly available from figshare).

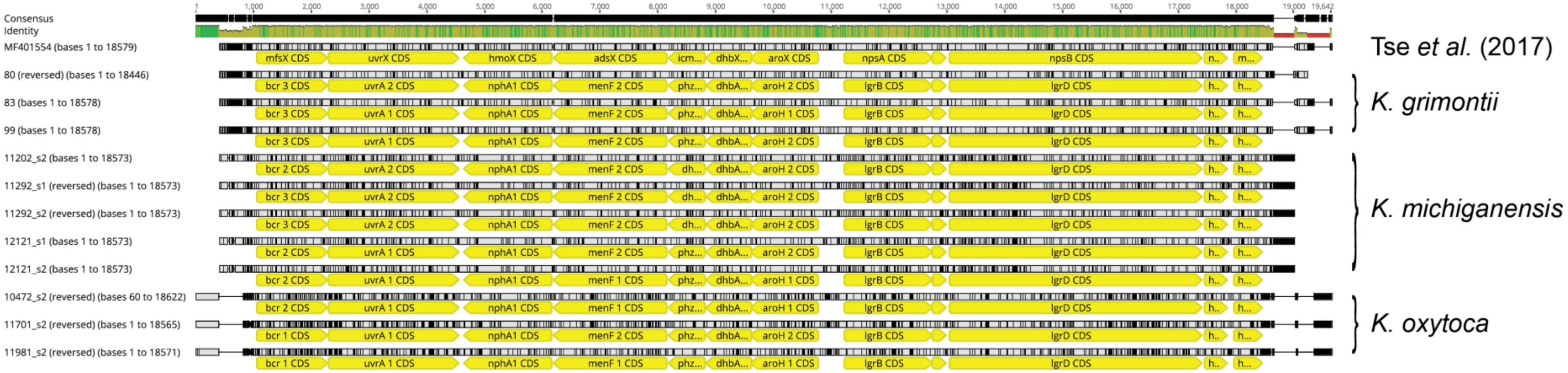

Supplementary Figure 5. Detection of the kleboxymycin BGC in strains and MAGs recovered from the faecal microbiota of preterm infants. The strains and MAGs were characterized by us previously (13). The image (alignment view) was generated via the progressiveMauve algorithm plugin of Geneious Prime v2019.2.1 (default settings, full alignment). Prokka-assigned gene annotations have been left for the infant-associated strains and MAGs. Two pairs of the *K. michiganensis* MAGs came from the same infants (11292, 12121) sampled at two different time points. Consensus identity is the mean pairwise nucleotide identity over all pairs in the column: green, 100 % identity; greeny-brown, at least 30 % and under 100 % identity; red, below 30 % identity.

REFERENCES

1. Public Health England. UK Standards for Microbiology Investigations P 8: Laboratory detection and reporting of bacteria with carbapenem-hydrolysing β-lactamases (carbapenemases) [Internet]. 2013. Available from: https://webarchive.nationalarchives.gov.uk/20140712120634/http://www.hpa.org.uk/webw/HPAweb&HPAwebStandard/HPAweb_C/1317138518829
2. EUCAST. Clinical breakpoints – bacteria v 5.0 [Internet]. 2015. Available from: http://www.eucast.org/fileadmin/src/media/PDFs/EUCAST_files/Breakpoint_tables/v_5.0_Breakpoint_Table_01.pdf
3. Bolger AM, Lohse M, Usadel B. Trimmomatic: a flexible trimmer for Illumina sequence data. Bioinforma Oxf Engl. 2014 Aug 1;30(15):2114–20.
4. Bankevich A, Nurk S, Antipov D, Gurevich AA, Dvorkin M, Kulikov AS, et al. SPAdes: a new genome assembly algorithm and its applications to single-cell sequencing. J Comput Biol J Comput Mol Cell Biol. 2012 May;19(5):455–77.
5. Parks DH, Imelfort M, Skennerton CT, Hugenholtz P, Tyson GW. CheckM: assessing the quality of microbial genomes recovered from isolates, single cells, and metagenomes. Genome Res. 2015 Jul;25(7):1043–55.
6. Seemann T. Prokka: rapid prokaryotic genome annotation. Bioinforma Oxf Engl. 2014 Jul 15;30(14):2068–9.
7. Jolley KA, Bray JE, Maiden MCJ. Open-access bacterial population genomics: BIGSdb software, the PubMLST.org website and their applications. Wellcome Open Res. 2018;3:124.
8. Lam M, Wyres KL, Duchêne S, Wick RR, Judd LM, Gan Y-H, et al. Population genomics of hypervirulent *Klebsiella pneumoniae* clonal-group 23 reveals early emergence and rapid global dissemination. Nat Commun [Internet]. 2018 Jul 13 [cited 2018 Aug 14];9. Available from: https://www.ncbi.nlm.nih.gov/pmc/articles/PMC6045662/
9. Wick RR, Heinz E, Holt KE, Wyres KL. Kaptive Web: user-friendly capsule and lipopolysaccharide serotype prediction for *Klebsiella* genomes. J Clin Microbiol. 2018 Jun;56(6).

10. Wyres KL, Wick RR, Gorrie C, Jenney A, Follador R, Thomson NR, et al. Identification of *Klebsiella* capsule synthesis loci from whole genome data. Microb Genomics. 2016 Dec;2(12):e000102.

11. Jia B, Raphenya AR, Alcock B, Waglechner N, Guo P, Tsang KK, et al. CARD 2017: expansion and model-centric curation of the comprehensive antibiotic resistance database. Nucleic Acids Res. 2017 Jan 4;45(D1):D566–73.

12. Liu B, Zheng D, Jin Q, Chen L, Yang J. VFDB 2019: a comparative pathogenomic platform with an interactive web interface. Nucleic Acids Res. 2019 Jan 8;47(D1):D687–92.

13. Chen Y, Brook TC, Soe CZ, O'Neill I, Alcon-Giner C, Leelastwattanagul O, et al. Preterm infants harbour diverse *Klebsiella* populations, including atypical species that encode and produce an array of antimicrobial resistance- and virulence-associated factors. Microb Genom. 2020 May 21;761924.

14. Hyatt D, Chen G-L, Locascio PF, Land ML, Larimer FW, Hauser LJ. Prodigal: prokaryotic gene recognition and translation initiation site identification. BMC Bioinformatics. 2010 Mar 8;11:119.

15. Segata N, Börnigen D, Morgan XC, Huttenhower C. PhyloPhlAn is a new method for improved phylogenetic and taxonomic placement of microbes. Nat Commun. 2013;4:2304.

16. Eades C, Davies F, Donaldson H, Hopkins K, Hill R, Otter J, et al. GES-5 carpabapenemase-producing *Klebsiella oxytoca* causing clinical infection in a UK haematopoetic stem cell transplantation unit. 26th European Congress of Clinical Microbiology and Infectious Diseases, Amsterdam, The Netherlands; 2016.

17. Merla C, Rodrigues C, Passet V, Corbella M, Thorpe HA, Kallonen TVS, et al. Description of *Klebsiella spallanzanii* sp. nov. and of *Klebsiella pasteurii* sp. nov. Front Microbiol. 2019;10:2360.

18. Ellington MJ, Davies F, Jauneikaite E, Hopkins KL, Turton JF, Adams G, et al. A multi-species cluster of GES-5 carbapenemase producing *Enterobacterales* linked by a geographically disseminated plasmid. Clin Infect Dis Off Publ Infect Dis Soc Am. 2019 Nov 20;

19. Sugumar M, Kumar KM, Manoharan A, Anbarasu A, Ramaiah S. Detection of OXA-1 β-lactamase gene of *Klebsiella pneumoniae* from blood stream infections (BSI) by conventional

PCR and in-silico analysis to understand the mechanism of OXA mediated resistance. PloS One. 2014;9(3):e91800.
